# A Preliminary Study of Neonatal Cranial Venous System by Color Doppler

**DOI:** 10.1155/2019/7569479

**Published:** 2019-04-28

**Authors:** Lu Yang Liu, Jin Ling Hong, Chang Jun Wu

**Affiliations:** First Affiliated Hospital of Harbin Medical University, 150001, No. 23 You Zheng Street, Harbin, Heilongjiang, China

## Abstract

**Aim:**

To present anatomic data in the ultrasound planes for the identification of the major veins and the venous sinuses in cerebrum and to establish the sonographic normal reference values for the visualization of vein vessels and vein sinuses and blood flow velocities.

**Methods:**

This study involved 55 healthy full-term neonates for transfontanellar color Doppler sonography. The imaging included both sagittal and coronal planes with LA332E probe, supplemented with PA240 probe as necessary. As low as reasonably achievable (ALARA) principle was obeyed, limiting Doppler exposure time and maximizing signal intensity by increasing gain rather than outputting transducer power settings. The output power was kept at a minimum level consistent with recording an adequate signal. Keeping the newborns in calm state, the total examination time which every neonate required was less than 5 min. All images were stored also in a workstation for further analysis. The description statistics and t-test for statistical analysis were used.

**Result:**

In all studied cases (100% cases), subependymal veins (SV), internal cerebral veins (ICV), Galen vein (GV), straight sinus (SS), superior sagittal sinus (SSS), and transverse sinuses (TS) were visualized. The visualization percentages of inferior sagittal sinus (ISS) or basal veins/Rosenthal veins (BV/RV) were lower than 100%. Based on vessel visualization percentage from high to low, the vessels were ordered as follows: SV, ICV, BV, SS, TS, ISS, and SSS. In SSS and TS, the pulsation percentage was 100%. The descending percentages of vessel pulsation were noted in SS, BV, ICV, and SV. On the basis of the mean of maximum velocities of the vessels from low to high, the vessels were ordered as follows: ISS, BV-L, BV-R, ICV-R, ICV-L, SV-L, SV-R, SSS, TS-L, TS-R, and SS.

**Conclusion:**

The measurements percent of visualization of cerebral deep veins was higher than the percent of cerebral venous sinuses. The pulsation percent of measurement and the velocities of cerebral venous sinuses were absolutely higher than the cerebral deep venous system. The pairs of vascular blood flow velocities were nonsignificantly different from one another.

## 1. Introduction

The intensive care of newborns has rapid advances. However, the substantial cases of neonatal illness and death due to brain injury are still globally reported each year [1]. The brain injury may be originated from trauma, hypoxia, and preterm delivery resulting in defects in the neuronal development. These defects are widely investigated by using cranial ultrasound [1].

Generally, an increase in the echogenicity is observed during the ultrasound examination through parasagittal view (posterior and superior to the ventricular trigone) of cranial region. The cranial sonograms of premature newborns commonly show the peritrigonal blush, i.e., a collection of fine, linear, and dense brush strokes. The second and third most prevailing brain injuries in neonates are periventricular leukomalacia (PVL) and germinal matrix or intraventricular hemorrhage (IVH), respectively. The neurologists consider PVL as the most occurring type of brain injury (3-10%) in the preterm newborns leading to various neurological conditions such as impaired vision, motor dysfunction, and cognitive impairment [2]. PVL sonography is based on an increase in white matter echogenicity with or without cyst formation [3]. There is a significant association between cystic PVL and neurological morbidity; therefore, it is critical to timely identify preterm neonate having the highest risk of consequent development of cystic PVL. Its prevention can be facilitated through early identification. The recently published studies [4, 5] have reported a decreased incidence of hemorrhage cases, likely due to the increased use of steroids during pregnancy and better care of neonates leading to the increased healthcare cost [6, 7]. These circumstances have raised the need of frequent screening pregnant females and the neonates through ultrasound. The recent studies have revealed spontaneous hemorrhage in the cerebral ventricle in both underweight (weighing < 500 g) and premature neonates (born before 35 weeks of gestational time) [8, 9]. The incidence of such cases is about 40 - 45% in neonates. Such type of injury is attributed to distortion of delicate capillaries present in the germinal matrix leading to hyperperfusion and periventricular infarction.

The parenchymal vascularity of neonatal brain is usually assessed by using conventional color Doppler sonography which involves the determination of frequency shifts and the direction of blood flow. A new evolution in the sonography is transcranial Doppler and transcranial color Doppler which are cost-effective, safe, portable, and noninvasive. Its transducer determines the rate of blood flow in Willis's loop by spectral analysis of frequency shifts after insonation with red blood cells flowing within a vessel. Transcranial Doppler is useful in determining the resistive index, peak systolic velocity, end diastolic velocity, and the mean velocity of blood flow within the studied duct [10].

Relatively little has been reported about the observations of newborn venous system with color Doppler ultrasound (CDU). These studies are still needed for better understanding of the unification of improving the accuracy of velocity measurements, the angle range between the vessel and the beam, and the precise placement of the sample volume for quantitative Doppler spectral analysis. Such studies would be helpful to quantitatively increase the structure visualization, evaluate the most suitable probes and frequencies, setting equipment, and so on [11–13].

This study involved 55 healthy term neonates who were born by cesarean section. The normal anatomy and the flow characteristics of intracranial venous system with CDU in newborns were analyzed in this study, revealing two major objectives such as (i) to present anatomic data in the ultrasound planes for the identification of the major veins and the venous sinuses in cerebral region, (ii) to establish sonographic normal reference values for the visualization of vein vessels and vein sinuses, and (iii) to determine the mean of maximum blood flow velocities.

## 2. Methods

### 2.1. Ethical Approval

The study was approved by the Ethics Committee of the First Affiliated Hospital of Harbin Medical University, and oral consent of the parents was obtained before all Doppler examinations.

### 2.2. Participants

The examination was performed at the bedside in the Maternity Ward of the First Affiliated Hospital of Harbin Medical University. This study involved the inclusion of 55 healthy full-term neonates (gestational age ≥ 37 weeks but < 42 weeks, birth weight ≥ 2500 g but < 4000 g, and postnatal age ≤ 2 days). During the period of examination, all infants remained stable, resting, lying supine.

### 2.3. Sonographic Technique

The ultrasound platform (MyLab25Gold, Esaote, Italy) with a 3.5-10.0 MHz Linear-Array transducer (LA332E) and a 2.0-3.5 MHz Phased-Array transducer (PA240) was used for all cases in this study. The spatial peak temporal average of the equipment estimated* in situ* was maintained below 94 mW/cm^2^.

The anterior fontanel approach had been used to obtain the conventional coronal views from the anteriormost to the posteriormost part. This was followed by side-to-side parasagittal sections. All neonates were examined with this approach. The imaging included both sagittal and coronal planes with LA332E probe, supplemented with PA240 probe as necessary.

As low as reasonably achievable (ALARA) principle was obeyed, limiting Doppler exposure time and maximizing signal intensity by increasing gain rather than outputting transducer power settings. The output power was kept at a minimum level consistent with recording an adequate signal [14–16]. The newborns were kept in calm or sleeping state during the examination. The total examination time that every neonate required was less than 5 min. All images were stored also in a workstation for further analysis.

### 2.4. Statistical Analysis

The description statistics and t-test with* p* < 0.05 were used for statistical analysis.

## 3. Results and Discussion

### 3.1. Scanning Approaches

The core purpose of this study was to introduce guidelines to analyze veins and veins sinuses and to perform a neonate cerebral veins examination within less than five minutes.

From the coronal views via anterior fontanel, a pair of subependymal vein, paired internal cerebral vein, a pair of basal vein, Galen vein, straight sinus, and paired transverse sinuses were approached. On the other hand, an internal cerebral vein, basal vein, Galen vein, inferior sagittal sinus, straight sinus, confluence, and superior sagittal sinus were observed from the sagittal views via anterior fontanel.

#### 3.1.1. Coronal Views via Anterior Fontanel Approach

Firstly, the frontal horns of lateral ventricles at the level of basal ganglia were easily found by using grayscale sonogram. Afterwards, color Doppler fontanel imaging was employed and found a pair of vessels along the inferolateral side of the frontal horns. The observed pair of vessels was the subependymal veins. The color of the two vessels were blue, and their pulsed-wave was flat and continuous.

The scans were performed along the subependymal veins, slightly posterior based on the original included angle (the referred angle was the one included between probe and front). The subependymal veins were close to midline medially. At the level where the third ventricle disappeared, the vessels appeared merging into the slightly bigger blue vessels which were on both sides of and parallel to the midline of the brain. They looked like “two pant tubes of a pair of trousers”: these were the internal cerebral veins.

The scans were continued along the internal cerebral veins, on the plane of body of lateral ventricle and identified the junction of the internal cerebral veins forming a bigger vein. This was the Galen vein which could be bright blue, bright red, or white. The diameter was wider and the length was shorter in comparison to internal cerebral veins.

In the near field of the junction of the internal cerebral veins forming the Galen vein, on both sides, the scans showed two dark red vessels passing around the cerebral peduncles and draining into junction of internal cerebral veins or vein of Galen; these were Rosenthal veins. The paired internal cerebral veins and the pair of Rosenthal veins looked like a pair of blue trousers and a pair of red shoes.

When the lateral ventricle choroid plexus disappeared, the scans identified a bright blue vessel, bigger than Galen vein, continuing coursing the flow from Galen vein and Rosenthal veins; this was the straight sinus, which ran towards the pillow part of skull.

**Figure 1:**
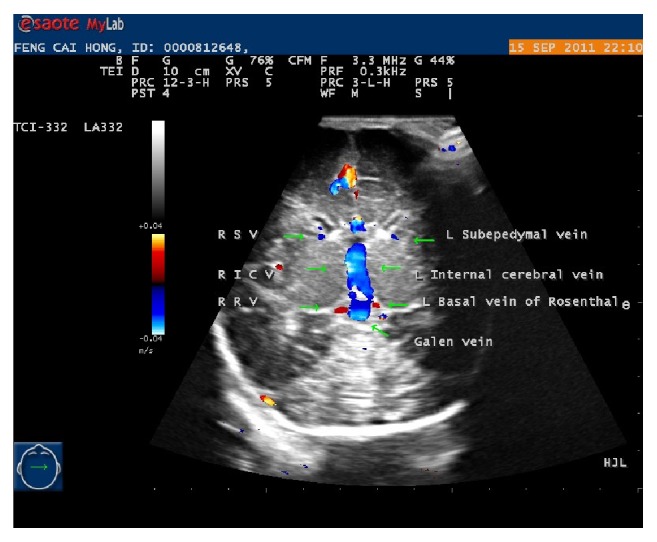
The color Doppler fontanel imaging of cranial venous system vessels at the coronal plane. The subependymal veins (SV), internal cerebral veins (ICV), Rosenthal veins (BV/RV), and Galen vein can be recognized from it.

Sometimes, the investigators saw a pair of vessels running separately on both sides of the straight sinus, close to the inside of skill. Those vessels were transverse sinuses. In most of the occasions, the color of both transverse sinuses was blue (Figure 1).

**Figure 2:**
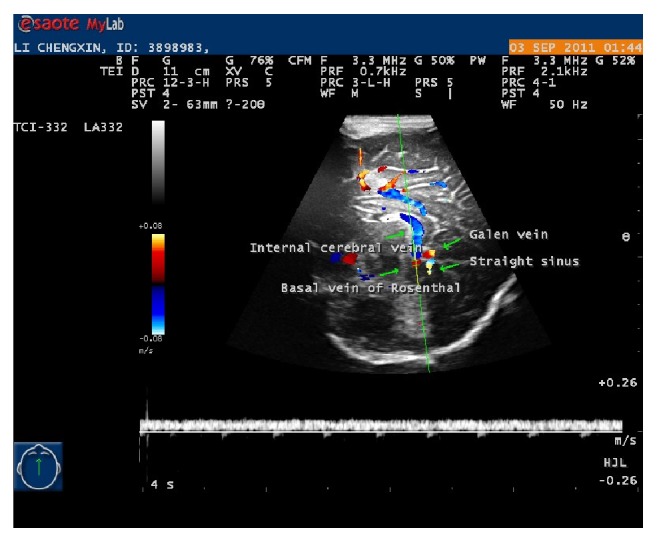
The transverse sinus at transverse plane.

As the probe was moved backwards, the transverse sinuses turned to be red because of the change of the positions in the anatomy. It indicates the evolution of sigmoid sinuses from transverse sinuses. The positions of transverse sinuses seemed almost symmetrical in ultrasound planes, but in fact not anatomically (Figure 2) and these findings are in accordance with literature [16–18].

#### 3.1.2. Sagittal Views through Anterior Fontanel

**Figure 3:**
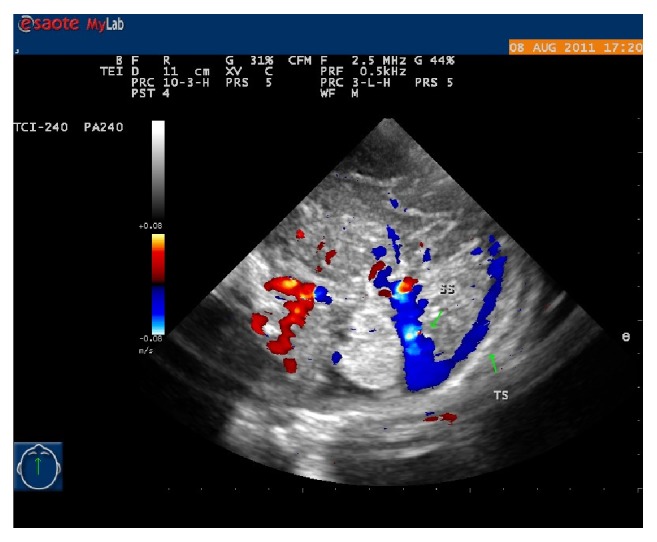
The brain midsagittal plane. The internal cerebral veins (ICV), straight sinus (SS), basal veins/Rosenthal veins (BV/RV), and Galen vein can be observed simultaneously.

**Figure 4:**
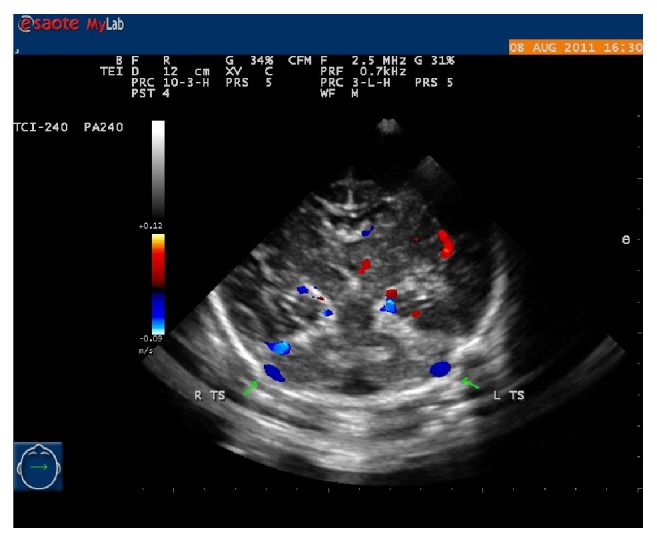
The brain Paramedian sagittal plane. The straight sinus (SS) and superior sagittal sinus (SSS) can be displayed.

At the midsagittal plane that might require a slight “twist” of the transducer from midline (to obtain a parasagittal-oblique view), a corpus callosum could be seen. Along the choroid plexus of the lateral ventricles, a blue vessel could be viewed (internal cerebral vein), running backwards below the corpus callosum splenium and draining into the Galen vein. Around the corpus callosum splenium, the internal cerebral vein became the Galen vein or great cerebral vein, which was curving sharply upwards and its color could be bright red or bright blue (Figure 3). The Galen vein was short, coursing upwards and then sharply downwards becoming straight sinus. The included angle between the Galen vein and straight sinus was less than 90°. The color of straight sinus was bright blue, and crossed above the cerebellum, and then drained into the confluence (Figure 4).

In the midsagittal plane and the inside of the occipital bone, there was a blue arched vessel: the superior sagittal sinus. In this position, the superior sagittal sinus was easy to visualize by a sector probe. The superior sagittal sinus could be distinguished clearly in all instances in the near field of the midline sagittal plane by a liner probe. The superior sagittal sinus drained to confluence as well.

Near the midsagittal plane, there were two more vessels near the junction of internal cerebral vein forming the Galen vein. One was inferior sagittal sinus, and another one was the Rosenthal vein.

Over the junction of internal cerebral vein draining into Galen vein, along the splenium of the corpus callosum, there was a small vessel [19, 20]: the inferior sagittal sinus. This was a small dark blue vessel with very low flow velocity, which was usually partially imaged on this median plane [21–23].

Below the junction of internal cerebral vein draining into Galen vein, near the thalamus, there was a small dark red vessel coursing upwards, passing around the cerebral peduncles and draining into Galen vein, which was Rosenthal vein [24, 25].

### 3.2. Observation Result

#### 3.2.1. Percentage Visualization

The visualized vessels were the paired subependymal vein, internal cerebral veins, Galen vein, straight sinus, superior sagittal sinus, and transverse sinuses (Table 1). The visualization of inferior sagittal sinus or pair of basal veins was lesser than 100%. The present results were consistent with the previous study [12]. The results of percentage visualization and pulsation reveal that the mean of maximum velocity of vessels was based on two intervals: the first interval's range was between 8.37 ± 3.95 cm/s and 11.46 ± 5.15 cm/s, while the second one ranged between 17.91 ± 8.77 cm/s and 20.60 ± 10.92 cm/s. The mean of maximum velocity of the paired subependymal veins, paired internal cerebral veins, and paired basal veins was nonsignificantly different (*p* > 0.05). The mean of maximum velocity of straight sinus, paired transverse sinuses, and superior sagittal sinus was nonsignificantly different (*p* > 0.05).

Compared with the mean of maximum velocity of the paired subependymal veins, paired internal cerebral vein, and paired transverse sinuses, there were nonstatistical differences (*p* > 0.05) and the result was consistent with another report [26]. There were only a few neonates who could get the velocity of the paired basal veins; thus the statistical comparison of the paired basal veins could not be established.

In 100% cases of vessels visualization, the paired subependymal veins and the paired internal cerebral veins were the easiest identifiable veins, since their anatomic positions were in near probe field. Compared with high percentage visualization of vessels, the visualization of inferior sagittal sinus or Rosenthal vein was lower. In CTV (computed tomographic venography) or MRV (magnetic resonance venography) examinations, the visualization percentage of inferior sagittal sinus or Rosenthal vein was lower than subependymal vein, internal cerebral vein, Galen vein (great cerebral vein), straight sinus, superior sagittal sinus, or transverse sinus [16, 19–23]. The present results were consistent with CTV and MRV examinations mentioned earlier. In present results, the percentage visualization of inferior sagittal sinus was lower (38.2%) compared to Taylor's result (50%) [12]. The inferior sagittal sinus, a small vessel with very low flow velocity, was usually partially imaged on this median plane [27]. The visualization of inferior sagittal sinus was highly variable [28]. The path of inferior sagittal sinus was above the splenium of the corpus callosum, and the scan revealed a blue vessel flowing along the corpus callosum and towards the splenium of the corpus callosum, where there might be an arterial signal, not a venous signal. The major disadvantage lied in the low-flow characteristics of a venous signal of the inferior sagittal sinus, which may be hidden by an arterial signal of the pericallosal artery [29].

The visualization percent of both posterior segments of basal veins (Rosenthal veins) was 16.3%. Only two neonates' basal veins could be seen who were studied by Taylor [12]. Although hypoplasia of Rosenthal vein seldom occurred, the variations were restricted to the posterior segments of basal vein. There was high insonation of basal vein that reflects a stable anatomy of the middle segment by TCD through a posterior transtemporal approach. However, the percent of visualization of basal vein by cerebral ultrasound was rather low, presumably due to its anatomic position and the variations of observation portion [30–32].

From the results of the percent of visualization, it can be concluded that through anterior fontanel, it was suitable to view the subependymal vein, internal cerebral vein, Galen vein (great cerebral vein), straight sinus, superior sagittal sinus, transverse sinus, and inferior sagittal sinus, not the Rosenthal vein.

#### 3.2.2. Measurement Percentage of Visualization

The position of vessels coursed near the bones of skull, and the included angle between vessels and beam from probe of insonation was too large especially if the neonate cried or moved unexpectedly during the pulsed-wave time. All the situations mentioned above could lead to the pattern of pulsed-wave less favorable for measurement. These were the reasons why not all the velocities of the visualized vessels could be measured (Table 1).

The measurement percentage of visualization of straight sinus and the pair of transverse sinuses were lower than the pair of subependymal veins and pair of internal cerebral veins. The positions of the straight sinus and the paired transverse sinuses were far from the probe, and near the skull bones, which had a close relationship of their anatomic positions. The measurement percentage of visualization of the pair of transverse sinus was different: the measurement percentage of the right transverse sinus was higher than the left one. Because the transverse sinuses were often asymmetric, the right was hyperplastic than the left [33–35]. The measurement percentage of visualization of superior sagittal sinus was the lowest in 100% cases of vessels' visualization. The superior sagittal sinus went along the upper margin of the falx and joined into the torcular herophili. The included angle between the superior sagittal sinus and the beam from the probe was very large [36–38]; therefore, the measurement percent of the superior sagittal sinus was the lowest.

#### 3.2.3. Mean of the Maximum Velocities and the Exclusion of Galen Vein and Torcular from Velocity Measurement

Generally speaking, the velocity of each vessel or sinus was higher than that documented previously (Table 1) [39–43], probably due to the higher precision of equipment used in the present study.

Akhtar and his colleagues indicated that the skull prevents any action of the atmospheric pressure on intracranial venous haemodynamics in adult [44]. For newborns, the incomplete closure of the skull could affect the venous flow in consequence of the influence of the atmospheric pressure on breathing. In this study, we put the newborns in a calm or sleeping state during the examination. At this time, the newborn's breathing is relatively smooth, and the influence of breathing is minimized as far as possible.

From the chart of the mean of the maximum velocities, the velocities of the vein sinuses were higher than that of the cerebral deep veins (the inferior sagittal sinus was a special sinus, because of its highly variable nature).

The data analysis suggests that the velocities have positive correlation with an increasing vessel size, thus reflecting the large amount of blood in the greater veins and sinuses. The similar results were reported in neonates and infants [45, 46].

Galen vein flow was nearly perpendicular to the beam from the probe. On the coronal and sagittal planes via the anterior fontanelle, the angle between the Galen vein and the ultrasound beam was almost perpendicular in this plane, frequency shift scan approached around zero, and Doppler signal was weak. Since the pair of internal cerebral veins, paired Rosenthal veins and other vessels drained into Galen vein, resulting in several branches in Galen vein. To measure the flow velocity, putting the sample volume close to branches having turbulence should be avoided. Therefore, Galen vein was not fit to measure velocity [39].

The torcular/confluence of sinuses, near the internal occipital protuberance, had an aliasing effect due to the turbulent flow coming from the straight sinus and the superior sagittal sinus, and its anatomic structure was not stable [40] and thus had no measurement significance.

#### 3.2.4. Pulsation Percent of Measurement

From the observed results (Table 1), most of the Doppler patterns of deep cerebral venous system were continuous flat, only a few with lower amplitude, in contrast most of the cerebral venous sinuses had palatial patterns, and the amplitudes were higher than the patterns of cerebral venous system. Parts of venous sinus flow velocity waveform were consisting of triphasic forward flow component. The present findings were consistent with those of other reports in the literature [12, 47, 48].

Whether cerebral venous flow was influenced by heartbeat, arterial vessels pulsation, or brain pulsations [12, 46], it was reasonable to suggest that the amplitude of venous pulsations was increased from the distal to the proximal portion of cerebral venous circulation.

A case of fourth degree intracranial hemorrhage was observed. Its scan showed a flat pattern of superior sagittal sinus. It can be attributed to the fourth degree intracranial hemorrhage leading to an encephaledema zone around it, which caused a rising intracranial pressure. The superior sagittal sinus could be compressed by a high intracranial pressure, and then the waveform was changed. From this case, it was also observed that the velocities of some of the deep venous system were lower than normal. The case report suggested that it was whether a common phenomenon or not in the cerebral edema in neonates showing the changes of the velocities or the waveform patterns. A previous study reported the cerebral venous waveforms altered with the rise in cerebral pressure, which could indicate a precise or earlier intracranial pressure changes than arterial waveform patterns in fetus [49]. The cerebral venous velocities and waveforms changes in cerebral edema in neonate may require further investigation.

Color Doppler was not as sensitive as CTV and MRV to observe the accurate position of venous anatomical variations at the edge field, just as the superior sagittal sinus and paired transverse sinuses (in the study, the paired transverse sinuses may be included in some occipital sinus [50]), but sensitively observing the vessels draining in the near field. It reveals that the vessels diameter could not be measured directly by color Doppler; however, an indirect measurement of the vessel diameters through the color Doppler signals is feasible [51].

## 4. Conclusion

This study about the neonatal cerebral venous vessels filled the gap in the neonatal cerebral haemodynamics. Nowadays, the technical advances of the newer Doppler instruments allowing optimization of sample volume, filter setting, and power intensity have facilitated the examination of the venous system which is providing more details and information than before. This study did not involve the* insonation of the petrosus sinuses, which in adults can be seen through the trans-condylar approach* [52]. Further studies can be conducted in future to discover more useful information in this field.

## Figures and Tables

**Table 1:** Hemodynamic features of neonatal cerebral veins and venous sinuses.

Vessel		Percent of visualization	Measurement		V_max_ (cm/s)
			Percent of visualization	Percent of pulsation	
Subependymal vein	Left	100% (55)	89.09% (49/55)	6.12% (3/49)	10.86 ± 4.94
	Right	100% (55)	85.45% (47/55)	6.38% (3/47)	11.46 ± 5.15

Internal cerebral vein	Left	100% (55)	85.45% (47/55)	17.02% (8/47)	10.23 ± 5.56
	Right	100% (55)	89.09% (49/55)	16.32% (8/49)	10.06 ± 4.74

Basal vein	Left	16.36% (9)	66.67% (6/9)	16.67% (1/6)	8.75 ± 5.24
	Right	16.36% (9)	88.89% (8/9)	12.50% (1/8)	8.69 ± 6.18

Inferior sagittal sinus		38.18% (21)	57.14% (12/21)	16.67% (2/12)	8.37 ± 3.95

Straight sinus		100% (55)	76.36% (42/55)	85.71% (36/42)	20.60 ± 10.92

Superior sagittal sinus		100% (55)	38.18% (21/55)	100% (21/21)	17.91 ± 8.77

Transverse sinus	Left	100% (55)	65.45% (36/55)	100% (36/36)	18.66 ± 7.55
	Right	100% (55)	78.18% (43/55)	100% (43/43)	19.52 ± 7.99

Galen vein /great cerebral vein		100% (55)	/	/	/

Confluence		100% (55)	/	/	/

## Data Availability

Data is not available.
